# Notes and Comments

**Published:** 1900-12

**Authors:** 


### Notes and Comments.[1]

AMALGAM ALLOYS.—In his characteristic style, Dr. J. Foster Flagg has given the profession another one of his thoughtful essays on the subject of amalgams.

"Truly it would seem," the doctor says, "as though a dental P. T. Barnum might have arisen in our midst, and that Joyce Heth, 'The Only Perfect' nurse of the 'Immortal Father of his Country,' together with 'The Woolly Horse' and 'The Happy Family,' were making for dentistry its 'Greatest Show on Earth;' and this has been going on for the past three years. . . . I have taught for many years that, as the profits to the supply-houses upon amalgam alloys were incredibly enormous, and that as dentists were paying exorbitant prices for materials which should be afforded them at reasonable rates, it ought to be that the charges for alloys should be no more than *half* that which they were."

---

A REPLY TO "AN UNJUST CRITICISM."—In reference to our recent note in this department upon "An Unjust Criticism," we are pleased to give the following from Dr. E. P. Beadles, of Danville, Va.:

"In a paper," the doctor says, "read in Baltimore, I used the words, 'Dentists are the greatest braggarts on earth,' etc. I do not think this can be construed to mean that *all* dentists are brag-

---

[1] The assistant editor solicits contributions for this department,—new methods, new remedies and formulas, or any short practical note which may prove of value to the practitioner or student. Address 1338 Walnut Street, Philadelphia.

garts; at any rate, I did not mean that the sentence should be so understood. I assert, however, that as a class there are very many braggarts among us, and that this thing works greatly to our hurt in many ways. When said, I was giving this as one reason why there is such an influx of young men into the dental profession. From our own statements, they regard us as great money-makers. I have it from good authority that not one-half of the dentists of the United States are able to pay their debts. I have heard otherwise excellent men continually bragging of how busy they are and how much they make."

